# Green Synthesis of Silver Nanoparticles Using the Leaf Extract of the Medicinal Plant, *Uvaria narum* and Its Antibacterial, Antiangiogenic, Anticancer and Catalytic Properties

**DOI:** 10.3390/antibiotics12030564

**Published:** 2023-03-13

**Authors:** Anthyalam Parambil Ajaykumar, Anjaly Mathew, Ayanam Parambath Chandni, Sudhir Rama Varma, Kodangattil Narayanan Jayaraj, Ovungal Sabira, Vazhanthodi Abdul Rasheed, Valiyaparambil Sivadasan Binitha, Thangaraj Raja Swaminathan, Valaparambil Saidumohammad Basheer, Suvendu Giri, Suvro Chatterjee

**Affiliations:** 1 Division of Bio-Nanomaterial, Department of Zoology, Sree Neelakanta Government Sanskrit College, Palakkad 679303, India; 2 Department of Chemistry, Sree Neelakanta Government Sanskrit College, Kerala 679303, India; anjalymathew4@gmail.com (A.M.);; 3 Clinical Sciences Department, Centre for Medical and Bio-Allied Health Sciences Research, Ajman University, Ajman P.O. Box 346, United Arab Emirates; 4 Basic Sciences Department, Centre for Medical and Bio-Allied Health Sciences Research, Ajman University, Ajman P.O. Box 346, United Arab Emirates; 5 Department of Zoology, Sree Narayana College, Kerala 680566, India; 6 Peninsular Marine Fish Genetic Resources Centre, ICAR National Bureau of Fish Genetic Resources (NBFGR), ICAR CMFRI Campus, Kochi 682018, India; 7 Vascular Biology Lab, AU-KBC Research Centre, MIT Campus of Anna University, Chennai 600025, India

**Keywords:** *Uvaria narum*, antiangiogenic activity, antibacterial activity, anticancer property, catalytic property, silver nanoparticles

## Abstract

Silver nanoparticles (AgNPs) made by green synthesis offer a variety of biochemical properties and are an excellent alternative to traditional medications due to their low cost. In the current study, we synthesised AgNPs from the leaf extract of the medicinal plant *Uvaria narum*, commonly called narumpanal. The nanoparticles were characterised by ultraviolet-visible (UV-Vis) spectroscopy, Fourier transform infrared spectroscopy (FTIR), scanning electron microscopy (SEM) and transmission electron microscopy (TEM). SEM analysis showed AgNPs are highly crystalline and spherical with an average diameter of 7.13 nm. The outstanding catalytic activity of AgNPs was demonstrated by employing the reduction of 4-nitrophenol to 4-aminophenol. The AgNPs showed antiangiogenic activity in the chick chorioallantoic membrane (CAM) assay. AgNPs demonstrated anticancer activity against Dalton’s lymphoma ascites cells (DLA cells) in trypan blue assay and cytotoxicity against three fish cell lines: *Oreochromis niloticus* liver (onlL; National Repository of Fish Cell Lines, India (NRFC) Accession number—NRFC052) cells, *Cyprinus carpio* koi fin (CCKF; NRFC Accession number—NRFC007) cells and *Cyprinus carpio* gill (CyCKG; NRFC Accession number—NRFC064). Furthermore, the AgNPs demonstrated their ability to inhibit pathogenic microorganisms, *Staphylococcus aureus*, and *Escherichia coli.* The results from the study displayed green synthesised AgNPs exhibit antiangiogenic activity, cytotoxicity, antimicrobial and catalytic properties, which are crucial characteristics of a molecule with excellent clinical applications.

## 1. Introduction

Today we are living in an era of nanoscience, which has a significant role in all spheres of life. Silver nanoparticles (AgNPs) are studied extensively when compared with other noble metal nanoparticles due to their optical, antimicrobial, anticancer, antioxidant and larvicidal properties, as well as their affordability [1,2,3]. In addition, they have a wide range of applications in sensors, catalysis and solar cells [4,5,6]. Various methods can be used for the synthesis of AgNPs; the most widely used route is the chemical reduction of Ag^+^ ions from an aqueous solution of silver nitrate and employing different reducing agents, such as ascorbic acid, hydrazine, ammonium formate, dimethylformamide and sodium borohydride [7,8,9,10,11]. Although the chemical reduction method is rapid, it is toxic and has a negative impact on the environment. Since green synthesis is eco-friendly, non-toxic and compatible with green chemistry protocols, we have chosen to concentrate on it as it opens up a wide range of current nanoscience research. A major drawback of phytochemical-mediated nanoparticle synthesis is that it requires relatively longer reaction times than conventional chemical reduction. This constraint can be overcome by coupling plant-mediated synthesis with microwave (MW)-assisted synthesis. MW-assisted chemical transformations are pollution-free, eco-friendly and offer high yields together with ease of processing and handling [12]. Numerous reports are available in the literature describing the plant-mediated synthesis of nanoparticles using MW-assisted methods [13,14,15].

In the current study, we report the synthesis of AgNPs using a simple, rapid and green technology using the leaf extract of *Uvaria narum*, a large woody climber (Family: Annonaceae), which is found in the hilly regions of Kerala, India. Phytochemical screening of the *U. narum* leaf extract by Achuthan and co-workers found that the leaves are rich in alkaloids, phenol, flavonoids, phytosterols and terpenoids. The same group also investigated the cytotoxic effect of the leaf extract of *U. narum* [16]. Investigations on the phyto-pharmacognostic features of *U. narum* leaf extract reveal that there is only a single layer of palisade cells under the upper epidermis. The space between the collenchyma cells and the vascular bundle is filled with parenchymatous cells, some of which contain calcium oxalate crystals that are both rosette- and prism-shaped. HPTLC fingerprint analyses of alcoholic and aqueous extracts confirmed the presence of quercitin as a biomarker polyphenolic component [17]. A recent biochemical study on *U. narum* has shown that it contains significant amounts of phenols, tannins, and antioxidants. The chemical composition of the plant reveals that the root bark has considerable concentrations of stereoisomers and acetogenins. Beta-sitosterol, glutinone, glutinol, taraxerol, and benzyl benzoate were also discovered from its leaf extract [18].

Due to the possible potency of phytochemicals in the leaves, the target of the present investigation was to explore the utilization of leaf extract of *U. narum* as a reducing agent and stabilising agent in AgNPs synthesis. The use of water in the extraction and reaction medium is a further add-on to green chemistry protocols. The catalytic activity of the biosynthesised AgNPs has also been tested by taking the reduction of 4-nitrophenol to 4-aminophenol in aqueous medium at room temperature as the model reaction. Antiangiogenic capabilities, antibacterial effects, anticancer characteristics and cytotoxic effects in three fish cell lines were investigated using the AgNPs synthesised using the leaf extract of *U. narum*.

## 2. Materials and Methods

### 2.1. Chemicals

Silver nitrate, sodium borohydride, *p*-nitrophenol were obtained from (Merck Chemicals Ltd., Mumbai, India). LG iWave microwave oven (Model 1S2021CW 2450 MHz, LG Electronics, Seoul, Republic of Korea) was used for extracting the phytochemicals from *U. narum* leaf in water and also for the synthesis of AgNPs. 

### 2.2. Preparation of U. narum Leaf Extract

**Figure 1:**
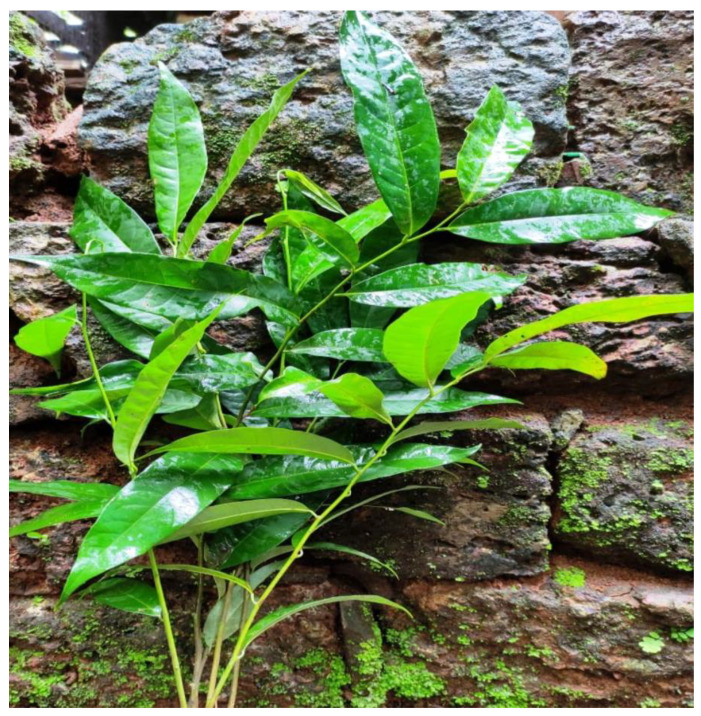
Photograph of *Uvaria narum* plant.

Fresh leaves of *U. narum* (Figure 1) were collected locally from the Malappuram (75°58′25.7″ E) district, Kerala state, India, and were identified with the help of an expert from the Department of Botany, University of Calicut, India. The leaves were washed thoroughly multiple times in deionised water and 10 g of the chopped leaves were taken in a 250-mL beaker and 200 mL distilled water was added. The beaker was then subjected to MW irradiation for about 2 min at a MW power of 350 W. The filtrate was then collected using Whatman filter paper and stored at 5 °C in a refrigerator for further studies.

### 2.3. Synthesis of AgNPs

AgNPs were prepared by heating a solution of AgNO_3_ and *U. narum* leaf extract in a domestic MW oven. In a typical procedure, 10 mL of the leaf extract was added to 50 mL of 0.001 M solution of silver nitrate and the reaction mixture was then placed in a domestic microwave oven at 2.45 GHz at 350 W for about 5 min. A change in colour, from colourless to light yellow and then to dark brown indicating the formation of AgNPs, was observed. The whole procedure was observed by recording the ultraviolet-visible (UV-Vis) absorption spectrum of the reaction mixture collected at regular intervals.

### 2.4. Characterization of Green Synthesised AgNPs

UV-Vis spectra (Thermo Scientific Evolution 160 UV-Vis spectrometer) of the periodically collected reaction mixtures were recorded to observe the surface plasmon resonance peak. The UV-Vis spectrum of the periodically collected reaction mixture was recorded every minute to observe the surface plasmon resonance peak. Fourier transform infrared (FTIR) spectra of the vacuum-dried *U. narum* leaf extract were recorded in the range 4000–450 cm^−1^ using a PerkinElmer FTIR spectrometer to identify the functional groups involved in the stabilization of the AgNPs. The surface morphology was analysed using a scanning electron microscope (SEM; VEGA3 TESCAN). The size and shape of green synthesised nanoparticles were analysed by transmission electron microscopy (JEOLJEM-2100 microscope, Montgomery, AL, USA).

### 2.5. Catalytic Capacity Evaluation

The biosynthesised AgNPs are found to be an efficient heterogeneous catalyst [19]. The hydrogenation of 4-nitrophenol to 4-aminophenol was used to investigate catalytic action of the prepared AgNPs. Millimolar solution of p- nitro phenol (2 mL) and sodium borohydride (0.5 mL) was mixed in the ratio 1:40, and 0.5 mL of AgNPs was added (0.1 mg/mL). The reaction was monitored by taking the absorption peak in 1 min time intervals in the range of 200–600 nm at room temperature using a UV-Vis spectrophotometer (Thermo scientific evolution 160).

### 2.6. Cyclic Voltammetry Analysis

The electrochemical response of *U. narum* leaf extract was studied by cyclic voltammetry (CV). The CV was carried out in a digital Ivy Potentiostat (Model No. DY2000EN) furnished with DY 2000 software. A three-electrode electrochemical setup containing a glassy carbon electrode (GCE) as the working electrode, platinum wire as a counter electrode and a Ag/AgCl reference electrode for recording CV. The CV measurements were performed with *Uveria* leaf extract containing 0.1 M phosphate buffer solution (pH −7.4) and 0.1 M KCl as supporting electrolyte.

### 2.7. Chick Chorioallantoic Membrane Assay

For the in vivo chorioallantoic membrane CAM assay to examine the angiogenic property of the nanoparticles, fertilised leghorn eggs of Hamburger–Hamilton stage 1 (HH stage 1) were obtained from Poultry Research Centre in Chennai, India. The CAM assay was performed in Vascular Biology Laboratory, AU-KBC Research Centre, MIT Campus of Anna University, Chennai, India. Eggshells were cleansed to make them sterile and then stored in an incubator at 37 °C with a humidity of 55%. To expose the CAM, a small window was cut in the eggs after four days of incubation (HH stage 24). A disc of Whatman no.1 filter paper saturated in AgNPs synthesised using *U. narum* was placed on the CAM. To maintain proper control for the experiment, filter papers soaked in phosphate-buffered saline (PBS) and crude plant extract were utilised. Eggs were returned to the incubator after the photographs were captured at the starting site. Using a stereo microscope (Nade, NSZ-810) and a MagnusPro 3.7 camera, images of the CAM were taken every 2 h. AngioQuant software evaluated the images taken at 0, 4 and 8 h to determine the length, size and junctions of the blood vessel [20,21].

### 2.8. Cytotoxicity of AgNps in Cancer Cell Lines

Dalton’s lymphoma ascites (DLA) cells were used to evaluate the cytotoxicity of AgNps. The analysis was carried out at the Amala Cancer Research Institute, Thrissur, India. DLA cells were aspirated from the peritoneal cavity of tumour-induced mice, washed several times with normal saline and a viable cell suspension of 1106 cells per 0.1 mL was transferred to tubes containing various concentrations of AgNPs, viz 10, 20, 50, 100 and 200 µg. PBS was used to get the volume up to 1 mL. In the control group, the same concentrations of *U. narum* leaf extracts were used. The assay mixtures were then incubated for 3 h at 37 °C before mixing with 0.1 mL of 1% trypan blue. After 3 min the number of dead and living cells was counted with a haemocytometer. The percentage of cytotoxicity was calculated with the following formula:

Percentage of cytotoxicity = number of dead cells/number of live cells + number of dead cells × 100.

### 2.9. Cytotoxicity of AgNPs in Fish Cell Lines

The cytotoxic effect of green synthesized AgNPs was tested on three fish cell lines: *Oreochromis niloticus* liver (onlL; National repository of fish cell line, India (NRFC) Accession number—NRFC052) cells (Swaminathan et al., 2018), *Cyprinus carpio* koi fin (CCKF; NRFC Accession number—NRFC007) cells (Swaminathan et al., 2015) and *Cyprinus carpio* gill (CyCKG; NRFC Accession number—NRFC064) cells (maintained at Peninsular Marine Fish Genetic Resources (PMFGR) center, ICAR National Bureau of Fish Genetic Resources (ICAR NBFGR), Kochi, Kerala, India) as described previously (Fornelli et al., 2004). The analysis was carried out in the laboratory of PMFGR, ICAR NBFGR, Kochi, Kerala, India. About, 2 × 10^5^ onlL, CCKF and CyCKG cells were seeded into 6-well tissue culture plates (Nunc, Roskilde, Denmark; Catalogue number—TMO140652) and after reaching 75–85% confluency, the cells were incubated with a mixture of 100 µL of AgNP samples and 900 µL of fresh medium. After removing the medium, plant extract was also taken as a control. Each cell was incubated at 28 °C using Leibovitz’s L-15 (L-15) medium (Life Technologies, Carlsbad, CA, USA; Lot number—2085192; Catalogue number—11415–064, 500 mL) supplemented with 2% fetal bovine serum (FBS) (Life Technologies, Carlsbad, CA, USA; Lot number—42G829K; Catalogue number—10270–106, 500 mL) and the effects of the plant extract and nanoparticles on the cells were observed after 24 and 48 hrs. For negative controls, wells were inoculated with 0.1 ml of sterile Phosphate Buffer Saline (PBS) (Life Technologies, Carlsbad, CA, USA; Lot number—1967526; Catalogue number—14190–144, 500 mL). Plates were incubated at 28 °C then the morphological changes of the cells after incubation with the AgNp and plant extract were noted.

### 2.10. Antimicrobial Activity

The in vitro antibacterial activity of AgNPs was assessed using the agar well diffusion method against Gram-negative (*Escherichia Coli*) and Gram-positive (*Staphylococcus aureus*) bacteria. Using a sterile cotton swab, the bacteria strains were disseminated on Mueller–Hinton agar (MHA) (Merck, Frankfurter, Germany) plates. In the test, a sterile blank antimicrobial susceptibility disc was employed. Two different concentrations (10 and 20 µL) of AgNPs were loaded onto the disc for the experiment, leaf extract (20 µL) of *U. narum* was utilised as a control, and the antibiotic ampicillin was employed as a positive control. The Petri dish was incubated for 16 h at 37 °C. The zone of inhibition was analysed to determine the antibacterial effects of the samples applied.

## 3. Results and Discussion

### 3.1. UV-Vis Spectral Analysis

**Figure 2:**
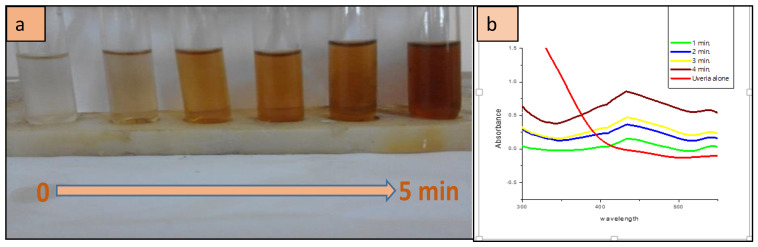
The colour change observed during MW exposure of AgNO_3_ (0.001 M) with *U. narum* leaf extract (**a**), UV-Vis spectra showing the effect of MW exposure over time (**b**) (Concentration of AgNO_3_: 0.001 M; MW power: 350 W).

*Uvaria narum* leaves are a rich source of alkaloids, phenol, flavonoids, phytosterols and terpenoids [17,18]. The water-soluble components of *U. narum* leaves caused the reduction of monovalent Ag^+^ ions to the corresponding metal nanoparticle. The change in colour of the reaction mixture, from colourless to dark brown upon MW irradiation, is a direct indication of the formation of the AgNPs (Figure 2a).

As the MW heating of the reaction medium having *U. narum* leaf extract and AgNO_3_ solution continues, the colour of the reaction medium changes from light-yellow (*t* = 1 min) to yellowish-brown (*t* = 3 min) and then to dark brown (*t* = 5 min), as indicated in Figure 2a.

UV-Visible analysis is a powerful tool for the characterisation of metal nanoparticles. Sharp surface plasmon absorption peaks are witnessed in the UV-Vis spectrum of noble metal nanoparticles because of the interaction of surface electrons with incident photons. The characteristics of each metal are determined by the size, shape and distribution of nanoparticles [22,23]. A typical plasmon resonance band at 427 nm is observed in the UV-Vis absorption spectra of the AgNPs synthesised using *U. narum* leaf extract which indicates the formation of AgNPs (the UV-Vis spectrum of leaf extract was also tested and it does not show any peak in the range 200–800 nm). The time-dependent UV-Vis absorption spectra are shown in Figure 2b. The intensity of the surface plasmon absorption band gradually increased on increasing the time of MW exposure and the maximum intensity of absorption was attained after 5 min of the reaction at a MW power of 350 W. It was known that compared with the other pathways, MW synthetic route produces narrow sized nanoparticles in a quick manner [21]. The reduction reaction behind the formation of nanoparticles can be written as follows:

$${AgNO}_{3}+phytochemicals\;in\;the\;leaf\;extract+Microwave\;irradiation\to AgNPs$$

From the results of the current study, it was found that the leaf extract of *U. narum* is an excellent reducing agent for the reduction of silver ions to silver. The phytochemicals in the leaf extract are powerful reducing agents for silver ions and stabilise the AgNPs.

### 3.2. FTIR Spectral Analysis

**Figure 3:**
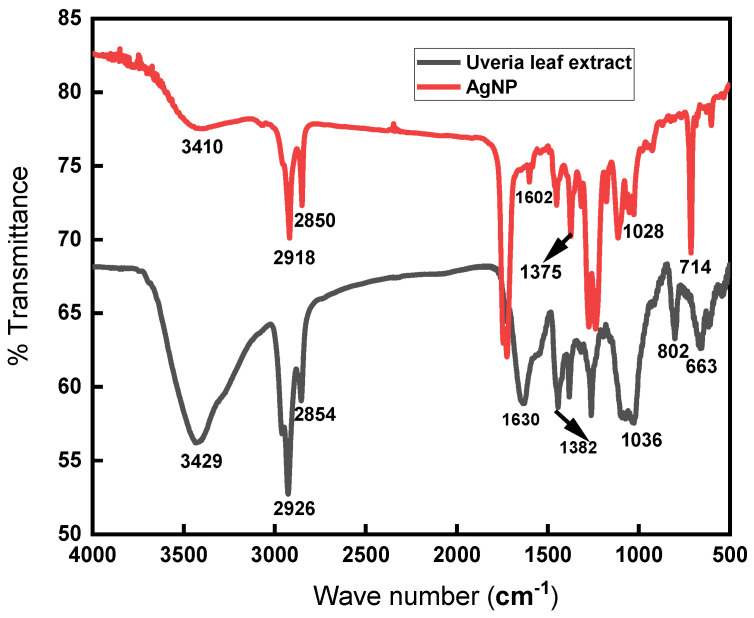
FTIR spectrum of biosynthesised AgNPs.

To recognise the possible functional groups involved in the production and stabilisation of the AgNPs, FT-IR spectral studies of the vacuum-dried *U. narum* leaf extract and AgNPs were carried out and are presented in Figure 3. FTIR spectrum of the *U. narum* leaf extract shows some distinct peaks corresponding to functional groups such as –OH, –NH, –CONH, –COOH and aromatic C=C linkages. The broad peak at 3429 cm^−1^ of the leaf extract is due to stretching vibrations of the phenolic O-H group that is hydrogen bonded. The peak at 2926 cm^−1^ and 2854 cm^−1^ corresponds to C-H stretching vibrations. The medium band at 1630 cm^−1^ corresponds to the amide I band. The peak around 1382 cm^−1^ is assigned to the –COO stretching from amino acid groups. The peak at 802 cm^−1^ is due to =C-H stretching (aromatic). This indicates that the terpenoids, flavonoids, phenols, and phytosterol found in the leaf extract of *U. narum* play a role in the formation of nanoparticles [18]. It can be inferred that biomolecules of leaf extract which contain active functional groups can reduce silver ions to metallic AgNPs. It is also proposed that these identified functional groups may interact with silver and form a capping layer over the AgNPs and thus prevent the aggregation of the nanoparticles. Previous studies have established that carbonyl, ester, amino, hydroxyl, and phenolic groups effectively bind with the noble metals through their polar and coordinating interactions [13]. The slight change in the intensity and position of the peaks in the spectrum of nanoparticles can be attributed to the coordination of phytochemicals with the metals [24].

### 3.3. SEM and TEM Analysis

**Figure 4:**
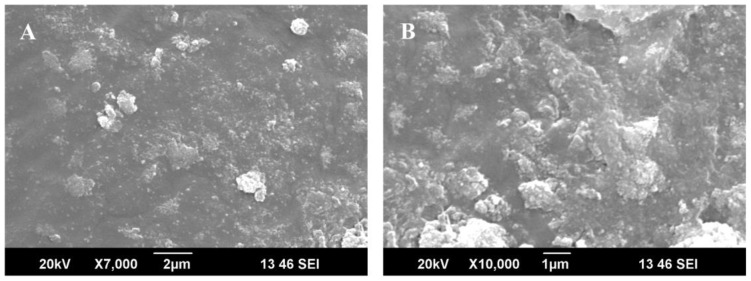
SEM images of the AgNPs prepared using *U. narum* leaf extract under different magnifications (**A**,**B**).

**Figure 5:**
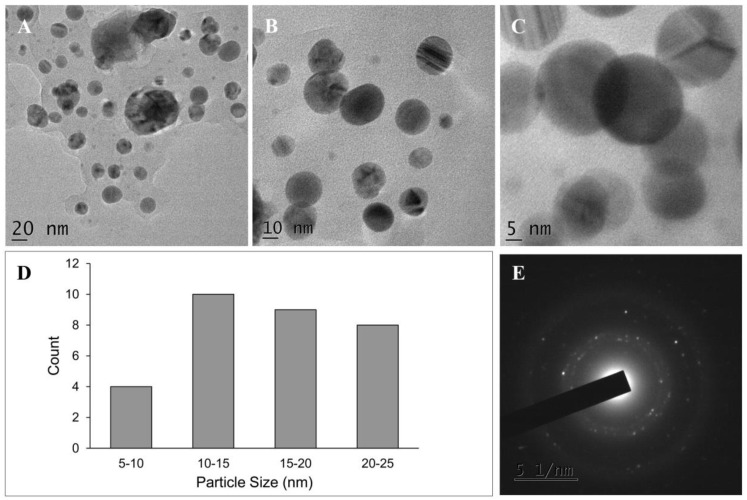
TEM images of the AgNPs under different magnifications (**A**–**C**), particle size histogram (**D**) and SAED pattern (**E**).

The surface morphology of synthesised AgNPs was studied by SEM and the images are shown in Figure 4. The size, morphology and crystal structure of green synthesised AgNPs were further studied in detail by TEM. As seen in Figure 5, it can be inferred that the AgNPs are almost spherical and monodisperse and size varies from 7 to 25 nm with an average size of 16.55 nm. The selected area electron diffraction (SAED) pattern shows bright circular rings which reveal the crystalline nature of the synthesised AgNPs (Figure 5E). The capping ability of the leaf extract is evident from the TEM images.

### 3.4. Catalytic Studies (Reduction of 4-Nitrophenol to 4-Aminophenol)

**Figure 6:**
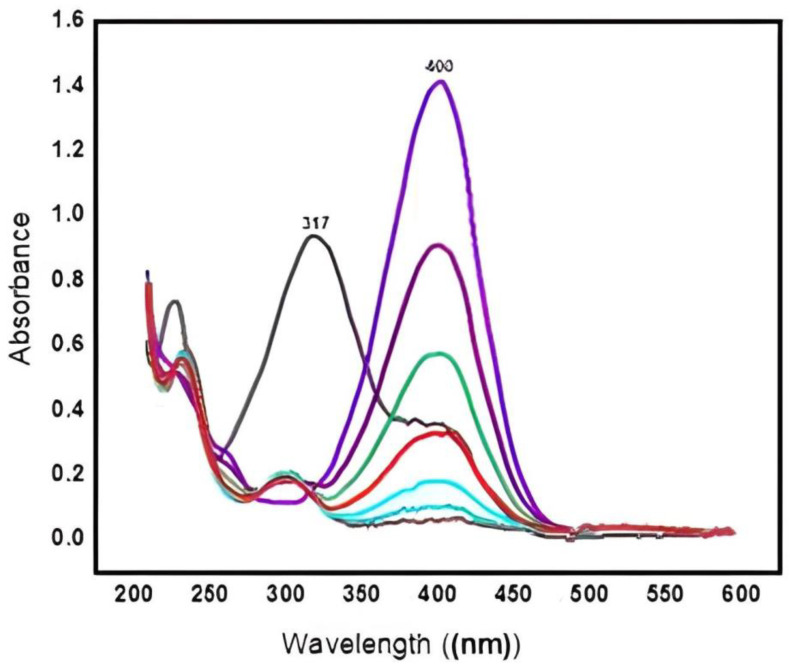
Time-dependant UV-Vis absorption spectra of NaBH_4_ reduction of 4-nirophenol. The graph indicates reduction of nitro-phenolate ion with time interval of 1 min.

The catalytic reduction of 4-nitrophenol (yellow coloured) to 4-aminophenol (colourless) in the presence of NaBH4 as a reducing agent was taken as a model reaction to evaluate the catalytic activity of green synthesised AgNPs. The aqueous solution of 4-nitrophenol is light yellow in colour and shows an absorption maximum at 317 nm. Upon the addition of NaBH_4_, the alkaline nature of the solution was increased and the corresponding phenolate ion was formed by hydrogen abstraction and the peak is shifted to 400 nm [23,24]. The peak at 400 nm rests for a long time without any change in its position indicating that, sodium borohydride alone is not sufficient for the reduction of 4-nitrophenol. With the addition of AgNPs, the red shift region at 400 nm dropped out and simultaneously appeared at the shoulder of the blue-shift wavelength, at 300 nm, which confirms the reduction of nitrophenolate ions to aminophenol, as shown in Figure 6 [25]. The catalytic reducing activity was monitored by UV-Vis spectral method at 1 min time intervals. The decrease in intensity of the peak at 404 nm is a direct indication of the reduction of 4-nitrophenol and reduction was observed within 8 min. This reaction proved that biosynthesized AgNPs have good catalytic activity in the reduction of 4-nitrophenol, providing a green and efficient catalyst for the reduction of organic pollutants in the environment.

The pollutant 4-nitrophenol is anthropogenic because of its toxic and inhibitory nature and is found in the industrial wastewater released from the manufacturing plants of pharmaceuticals, pesticides, dyes and papers [26,27]. On the other hand, its reduction product 4-aminophenol has several applications and is used for the synthesis of anticorrosion lubricants, antipyretic drugs, photographic developer, analgesics, etc [28]. Hence, the reduction of 4-nitrophenol to 4-amino phenol is very important. Although the reduction of 4-nitrophenol to 4-aminophenol using the reducing agent NaBH_4_ was a thermodynamically allowed reaction, it was kinetically forbidden, even for a few days, by NaBH_4_ alone [29].

### 3.5. CV Analysis

**Figure 7:**
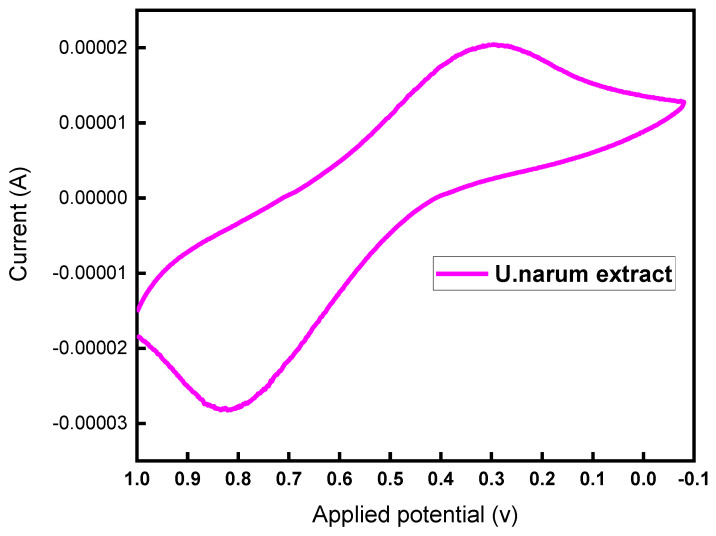
CV analysis of leaf extract of *U. narum*.

Cyclic voltametric measurements were performed to investigate the electrochemical response of *U. narum* leaf extract. The resultant cyclic voltagram is given in Figure 7. An oxidation peak is observed at a potential 0.297 V, which is greater than the potential of Ag/AgCl system (0.2224 V). CV analysis confirmed the effective reduction capacity of the *U. narum* leaves extract to reduce Ag ions to AgNPs, similar results were obtained using *Mimosa albida* leaf extract [30].

### 3.6. Antiangiogenic Assay

**Figure 8:**
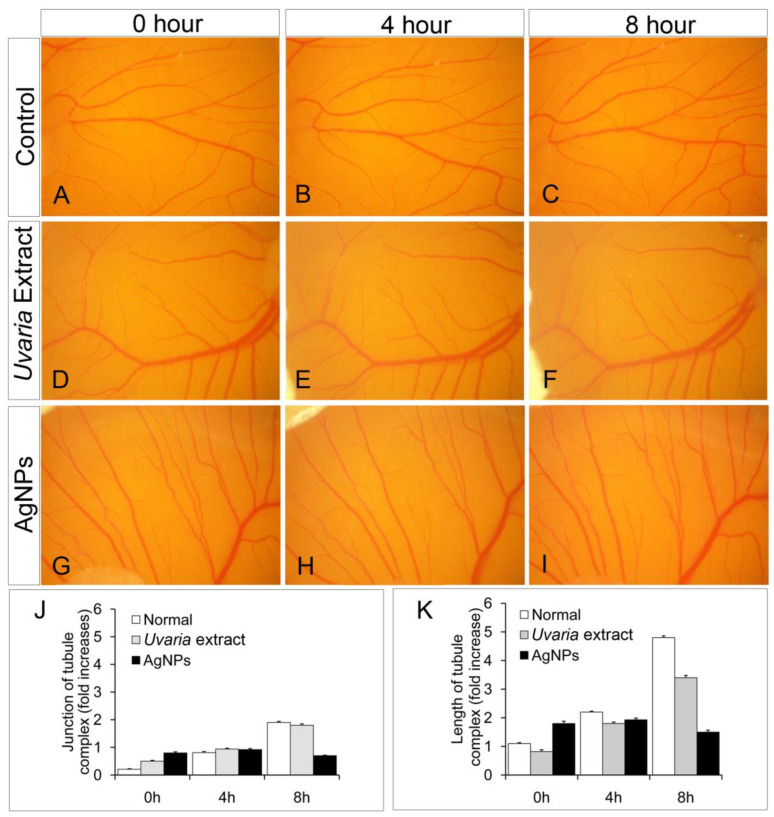
Antiangiogenic activity of AgNPs in vitro CAM assay: AgNPs inhibited the development of blood vessels demonstrating the antiangiogenic nature of the nanoparticles (**G**–**I**). Phosphate buffer (**A**–**C**) and leaf extracts (**D**–**F**) were used for comparison. The quantification of the images with respect to length and junction was analysed and represented as mean ± SD (**J**,**K**). AgNPs showed a significant reduction in blood vessel length and junction formation (*p* < 0.05).

The biosynthesised AgNPs were tested at various time intervals (0, 4 and 8 h) to analyse antiangiogenic effect. The data showed the inhibitory effects of AgNPs on blood vessel formation (Figure 8); however, the control and plant extract samples appeared to be normal. This obstructive effect indicates the considerable inhibitory effect of AgNPs in the formation of blood vessels.

‘Inducing angiogenesis’ is one of the hallmarks of cancer. Most tumours become heavily vascularised to aid tumour cell metabolism, and anti-angiogenic therapy can be considered a targeted therapeutic strategy in cancer. Silver nanoparticles have been demonstrated to inhibit angiogenesis [31]. Many other nanoparticle complexes have been demonstrated to exhibit anti-angiogenic activity [32,33,34]. In this study, we found the potency of anti-angiogenic activity of green synthesised AgNPs by employing the CAM assay through in-depth molecular mechanisms needs to be explored. Earlier investigations showed that the spherical shape of AgNPs significantly inhibits angiogenic activity [35]. Moreover, nanoparticles of size 20 nm appeared to be more effective molecules as they showed a binding of vascular endothelial growth factor (VEGF) that led to the inhibition of angiogenesis [36]. Thus, a spherical shape and nanoparticle size are most likely contributed to the antiangiogenic properties of the green synthesised AgNPs using *U. narum* leaf extract. Further receptor-based investigations are essential to draw a conclusion about the inhibitory effect of AgNPs on VEGF.

### 3.7. Anticancer Activity of AgNPs

**Figure 9:**
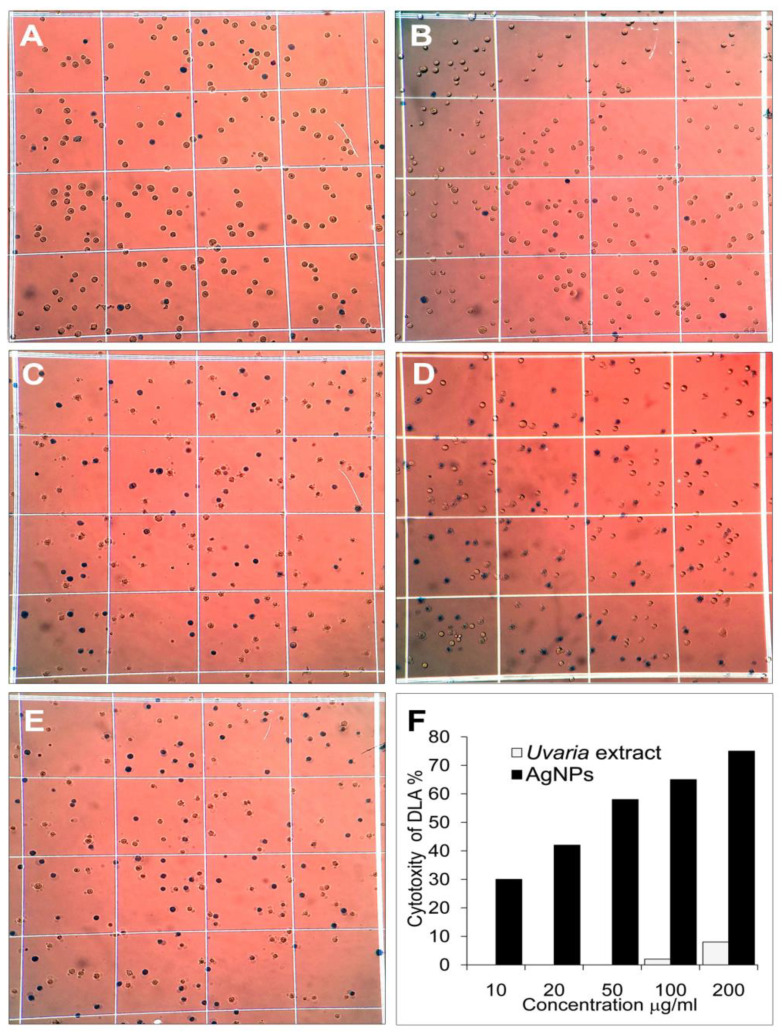
Analysis of in vitro cancer cell cytotoxicity of AgNPs (**A**–**E**). The quantification of anticancer activity was estimated and represented as bar diagram (**F**). The green synthesised AgNPs showed significant cytotoxicity against the control group.

The anticancer properties of green synthesised AgNPs were investigated with DLA cells. The results of the cytotoxic assay are presented in Figure 9. The data show that AgNPs reduce the viability of DLA cells in a dose-dependent manner. Similarly, AgNPs reduced the cell survival of MCF7 cell lines in vitro and could operate as a human breast cancer-controlling agent [37]. Bethu et al., (2018) found that biosynthesised AgNPs have concentration-dependent anticancer activity against the cancer cell lines SKOV3, DU145, PC-3 and A549 [38]. The anticancer property of biologically synthesized silver nanoparticles is assumed to be due to the activation of reactive oxygen species by the AgNPs, which leads to oxidative damage to cellular components like DNA, proteins, and lipids and ultimately cell death [39]. Numerous biomolecules, in particular, polyphenolic compounds from the leaf extracts of the *U. narum* adsorbed onto the surface of AgNPs, may be another reason for the anticancer activity. Thus, green synthesised AgNPs have been suggested as an effective molecule for controlling cancer cells in vitro.

### 3.8. Cytotoxicity Assay in Fish Cell Lines

**Figure 10:**
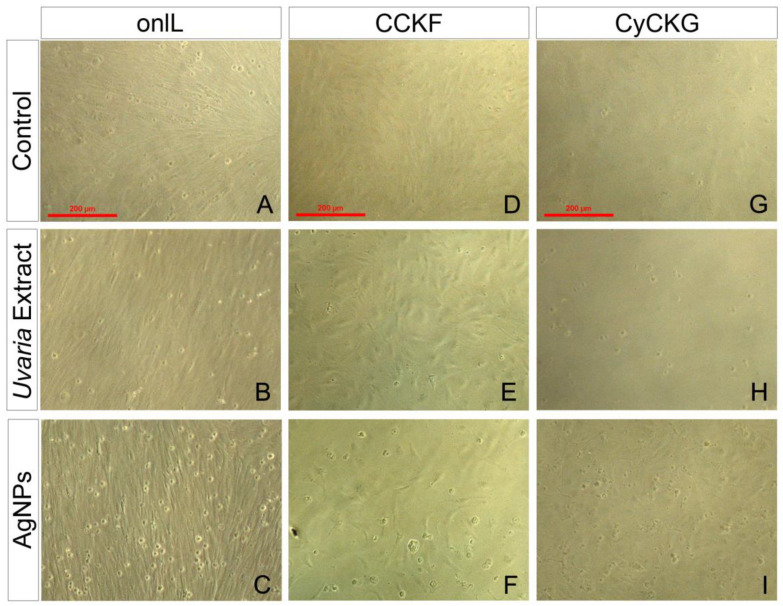
Cytoxicity of AgNPs as observed by morphological changes in three fish cell lines: onlL, CCKF and CyCKG. In comparison to plant extract (**B**,**E**,**H**) and control (**A**,**D**,**G**), green produced AgNPs demonstrated greater cytotoxicity (**C**,**F**,**I**) in the three fish cell lines.

Cytotoxicity of the silver nanoparticles was evaluated against three fish cell lines, onlL, CCKF, and CyCKG at different concentrations. Our results suggest a direct dose-response relationship with the tested cells at higher concentrations. Different morphological changes were observed in the spindle-shaped fibroblastic cells of all three fish cell lines treated with green synthesized AgNPs. The AgNPs produced an extensive cytotoxic effect, including cell shrinkage, rounding, and cell fusions in all three cell lines (Figure 10). The improved cytotoxic effects may be due to the presence of bioactive compounds as capping agents in the green synthesis of silver nanoparticles. Furthermore, a gradual increase in cytotoxicity could be seen (Figure 10) when increasing the concentration of AgNPs. Previous research has demonstrated nanoparticles can inhibit fish cell lines in a dose-dependent manner [40]. The toxicity of AgNPs has been previously studied in fish species such as Japanese medaka (*Oryzias latipes*) [41,42], zebrafish (*Danio rerio*) [43,44,45], rainbow trout (Oncorhynchus mykiss) [46] and sheephead minnow (*Cyprinodan variegatus*) [47] with an LC50 value in the range of 0.089–250 µg/mL. Experiments suggest that aquatic animals like fishes are highly susceptible to metal nanoparticles [48,49]. Evaluation of the metallic nanoparticles in the cell culture system is supported by ethical considerations and its adherence to the 3Rs’ principles of replacement, reduction, and refinement. Moreover, they are easy to maintain and able to obtain reproducible results. Similarly, in the present experiment, we could obtain consistent results across all three fish cell lines. Although there are numerous fish cell lines available from various species [50], they have been utilised in a limited number of studies to evaluate the toxicity of nanoparticles. However, these in vitro experiments using fish cell lines have demonstrated the cytotoxic effects of the metal nanoparticles [50,51,52,53,54]. Experiments in the rainbow trout cell line (RTL-W1) suggest that the composition of the cell culture media, particularly with high amino acid content, can significantly influence the behaviour and toxicity of AgNPs [54]. Thus, comprehensive precautionary measures that take fish cell lines into account need to be factored in during experiments using metallic nanoparticles. Moreover, careful attention should be given to exposure routes and potential target sites to ensure that the data obtained are meaningful when employing an in vitro approach.

### 3.9. Antimicrobial Activities

**Figure 11:**
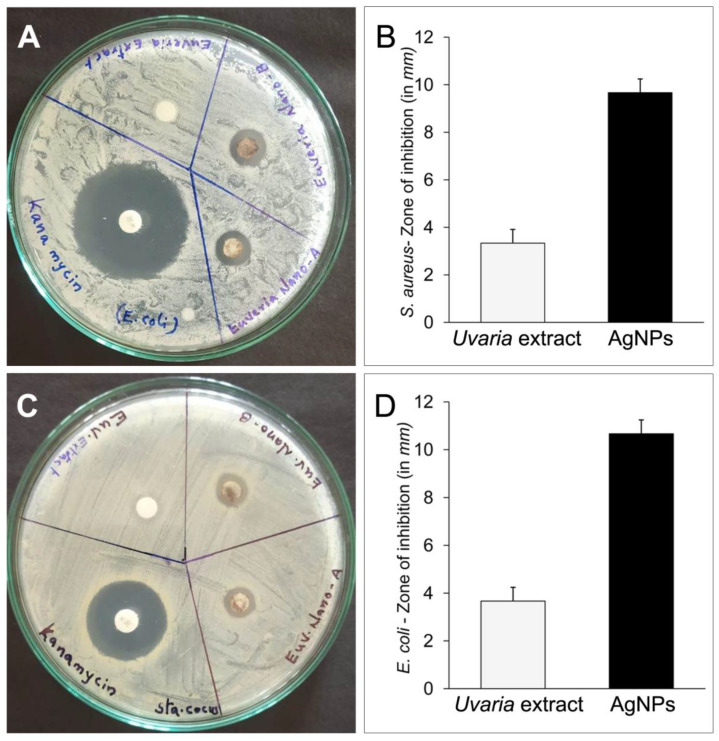
AgNPs showed a clear zone of inhibition against *E. coli* (**A**) and *S. Aureus* (**C**). Kanamycin (positive control) and *Uvaria* leaf extract were used as controls. The quantification of antimicrobial activity was estimated and represented as bar diagram (**B**,**D**).

Agar disc diffusion assay method was used to investigate the antibacterial properties of AgNPs in vitro. The findings revealed that green synthesised AgNPs had a considerable antibacterial activity (Figure 11). The zones of inhibition against *S. aureus* and *E. coli* were determined to be 9.6 ± 0.58 mm and 10.67 ± 0.58 mm, respectively. The leaf extracts (control group) showed modest antibacterial effects against *S. aureus* (3.33 ± 0.13 mm) and *E. coli* (3.67 ± 0.11 mm) [55,56]. This may be due to the presence of phytochemicals in the *U. narum* leaf extract. The synergistic interaction between AgNPs and natural chemicals in the leaf extract is one of the major reasons for the improved antibacterial activity of AgNPs, as demonstrated in an earlier study. The antibacterial activity of AgNPs is based on targeting the respiratory chain and cell division of bacteria, which eventually leads to cell death. It has also been found that AgNPs release silver ions inside bacterial cells, boosting their bactericidal effects [57].

## 4. Conclusions

The microwave-assisted synthesis of AgNPs using the leaves of the medicinal plant, *U. narum*, via a green chemistry approach has various benefits, such as an economical, energy-efficient and environmentally friendly process. The bioactive phytocompounds found in the aqueous extract of *U. narum* making them effective stabilising and capping agents. The formation of AgNPs was determined under UV-Vis Spectroscopy, monitored at 427 nm. FTIR spectral analysis verified the presence of the functional group of the bioactive components. The spherical shape and particle size of AgNPs were determined by TEM and SEM studies. The biosynthesised AgNPs showed their antibacterial activity against Gram-positive and Gram-negative bacteria as well as their antiangiogenic effects using CAM assay. AgNPs displayed cytotoxicity against fish cell lines and anticancer potential against Dalton’s lymphoma ascites cell lines. All these biochemical properties are crucial characteristics of a molecule with excellent biomedical applications. Additional in vivo bioassay-guided studies are required to determine the medicinal efficacy of the AgNPs synthesised using the leaf extract of the medicinal plant, *U. narum*.

## Data Availability

The data generated and analysed during the current study are available from the corresponding author upon reasonable request.
